# Contemporary Trends of the Epidemiology, Clinical Characteristics, and Resource Utilization of Necrotizing Fasciitis in Texas: A Population-Based Cohort Study

**DOI:** 10.1155/2015/618067

**Published:** 2015-03-29

**Authors:** Lavi Oud, Phillip Watkins

**Affiliations:** ^1^Division of Pulmonary and Critical Care Medicine, Department of Internal Medicine, Texas Tech University Health Sciences Center at the Permian Basin, 701 W. 5th Street, Odessa, TX 79763, USA; ^2^Clinical Research Institute, Texas Tech University HSC, 3601 4th Street, MS6238, Lubbock, TX 79430, USA

## Abstract

*Introduction*. There are limited population-level reports on the contemporary trends of the epidemiology, clinical features, resource utilization, and outcomes of necrotizing fasciitis (NF). *Methods*. We conducted a cohort study of Texas inpatient population, identifying hospitalizations with a diagnosis of NF during the years 2001–2010. The incidence, clinical features, resource utilization, and outcomes of NF hospitalizations were examined. *Results*. There were 12,172 NF hospitalizations during study period, with ICU admission in 50.3%. The incidence of NF rose 2.7%/year (*P* = 0.0001). Key changes between 2001-2002 and 2009-2010 included rising incidence of NF (5.9 versus 7.6 per 100,000 [*P* < 0.0001]), chronic comorbidities (69.4% versus 76.7% [*P* < 0.0001]), and development of ≥1 organ failure (28.5% versus 51.7% [*P* < 0.0001]). Inflation-adjusted hospital charges rose 37% (*P* < 0.0001). Hospital mortality (9.3%) remained unchanged during study period. Discharges to long-term care facilities rose from 12.2 to 30% (*P* < 0.0001). *Conclusions*. The present cohort of NF is the largest reported to date. There has been increasing incidence, chronic illness, and severity of illness of NF over the past decade, with half of NF hospitalizations admitted to ICU. Hospital mortality remained unchanged, while need for long-term care rose nearly 2.5-fold among survivors, suggesting increasing residual morbidity. The sources of the observed findings require further study.

## 1. Introduction

Necrotizing fasciitis (NF) is a rapidly progressive infection involving necrosis of the fascia and subcutaneous tissues [1]. Because NF occurs rarely, reports on its epidemiology, clinical features, resource utilization, and outcomes are commonly based on relatively small case series and cohorts, limiting generalization of reported findings [2, 3]. Delays in recognition and timely intervention in patients with NF are common and are associated with increased mortality [1]. Patients with NF can rapidly deteriorate, develop organ failure, and often require care in an intensive care unit [4], with case fatality that may exceed 40% [5].

Relatively few population-based studies of NF have been performed [6, 7], but they varied on reporting the incidence, associated organ dysfunction, used resources, and patient outcomes. There are no population-level data on the recent trends of the incidence, evolving organ failure, resource utilization, and outcomes of NF in the United States (US).

We sought to perform a population-based examination of the contemporary patterns of the epidemiology, clinical features, resource utilization, and outcomes of patients hospitalized with NF.

## 2. Materials and Methods

### 2.1. Data Sources

We used the Texas Inpatient Public Use Data File (TIPUDF), a longitudinal data set maintained by the Texas Department of State Health Services [8] to perform a retrospective, population-based investigation of necrotizing fasciitis. The data set includes detailed deidentified inpatient discharge data from all state-licensed hospitals, with the exception of those exempt by state statute from reporting to the Texas Health Care Information Collection. Exempt hospitals include (a) those that do not seek insurance payment or government reimbursement and (b) selected rural providers, based on beds number and local county population [8]. The facilities included in the mandated report account for 93% to 97% of all hospital discharges. The TIPUDF data set includes demographic, clinical, resource utilization, and outcome information. The data set includes up to 25 discharge diagnoses and up to 25 procedures, coded using the* International Classification of Diseases*,* Ninth Revision*,* Clinical Modification* (ICD-9-CM). US Census data of censal reports and intercensal population estimates were used to derive incidence and mortality rates [9]. In addition, US Census data were used to derive information on data on the median income and the proportion of residents living below poverty line of the population residing at the zip code of NF. Because we used a publicly available, deidentified data set, this study was determined to be exempt from formal review by the Texas Tech Health Sciences Center Institutional Review Board.

### 2.2. Study Population

We used an ICD-9-CM code 728.86 to identify state residents aged ≥15 years hospitalized with a primary or secondary diagnosis of necrotizing fasciitis during the years 2001–2010. The TIPUDF does not include data on the anatomical sites affected by NF (i.e., limbs, torso).

### 2.3. Data Collection

We collected data on patients' age, gender, race (categorized as non-Hispanic black (black), non-Hispanic white (white), Hispanic, and other), zip code at area of residence, and health insurance (categorized as private, Medicaid, Medicare, uninsured, and other); reported ICD-9-CM-based chronic comorbid conditions (based on the Deyo modification of the Charlson Comorbidity Index [10]), obesity, smoking, drug and alcohol abuse, other sites of infection (see Supplementary Table 1 in Supplementary Material available online at http://dx.doi.org/10.1155/2015/618067), type and number of failing organs (Supplementary Table 2), admission to an ICU (defined as presence of an Intensive Care Unit charge greater than $0), life-support interventions (mechanical ventilation, central venous catheterization, and hemodialysis; new hemodialysis was defined as a combination of hemodialysis code and absence of ICD-9-CM codes of chronic renal failure) (Supplementary Table 3), teaching status of the hospital, reported total hospital charges, hospital length of stay, and disposition at the end of hospitalization. Severity of illness was based on the number of failing organs (OF), modeled by the coding system reported by Lagu et al. [11].

### 2.4. Outcomes

The primary outcome was hospital mortality. Secondary outcomes included number and type of OF, resource utilization, and disposition among hospital survivors.

### 2.5. Data Analysis

Because TIPUDF provides discharge-level rather than patient-level information, we reported NF events as number of hospitalizations. In order to derive the incidence of NF hospitalizations, we used the direct method for age adjustment.

The mortality associated with NF was examined as both* hospital mortality* (defined as the number of NF hospitalizations who died in the hospital divided by the total number of NF hospitalizations for an examined group) and* morality rate* per 100,000 population.

Because changes in the frequency of reported organ failures over time may represent overcoding [11], we compared the rates of utilization of organ-specific life-support interventions among NF hospitalizations with a specific organ failure (i.e., use of mechanical ventilation among hospitalizations with reported respiratory failure) at the start and end of study period.

Group data are reported as numbers (percentages) for categorical variables and mean (standard deviation (SD)) or median (interquartile range (IQR)) for continuous variables, as appropriate. Ninety-five percent confidence intervals (95% CI) were calculated. Distribution of normality was examined by Kolmogorov-Smirnov test. Categorical data were compared by a 2-sided *χ* ^2^ test. Mann-Whitney *U* test and *t*-test were used to compare continuous data, as appropriate. We used regression analysis to explore the annual trends of NF incidence, hospital mortality, hospital length of stay, and hospital charges. When examining changes of key cohort characteristics at the start versus end of past decade, we used combined 2-year data to enhance precision of comparisons. Total hospital charges were examined using inflation-adjusted (2010) dollars [12]. Multivariate logistic regression models were constructed to examine candidate covariates as predictors of NF-associated hospital mortality. Covariates were considered for multivariate regression models if they either were statistically significant (*P* < 0.05) or had odds ratio ≥1.5 or ≤0.66 on univariate analysis. Candidate covariates included age, gender, race/ethnicity, health insurance, level of poverty and median income at the zip code of residence, chronic comorbidities, obesity, smoking, drug or ethanol abuse, teaching status of hospitals, type and number of failing organs, use of selected life-support interventions, and use of hyperbaric oxygen. The impact of the type and number of failing organs was analyzed in separate models. Because the state of Texas masks zip code and gender data of hospitalizations with a diagnosis of infection with the human immunodeficiency virus (HIV), ethanol or drug abuse, analyses of the role of gender, and the level of local poverty or median income were restricted to NF hospitalizations without the aforementioned 3 diagnoses. Similarly, models that examined the predictive role of HIV infection and ethanol or drug abuse did not include gender and the level of local poverty or median income.

All statistical analyses were performed using MedCalc version 12.7.0 (MedCalc Software, Ostend, Belgium) and SAS version 9.3 (SAS Institute, Cary, NC, USA). A 2-sided *P* value <0.05 was considered statistically significant.

## 3. Results

There were 21,983,203 hospitalizations aged ≥15 years between 2001 and 2010, of which 12,172 had reported NF. The characteristics of NF hospitalizations are detailed in Table 1. Most NF hospitalizations (75.8%) were younger than 65 years, and 54% were male. Nearly 1 in 5 NF hospitalizations (19%) lacked health insurance. The majority (72.2%) had one or more chronic comorbidities, with diabetes (47.6%) being the most common. Obesity was reported in 15.2%. Reported drug abuse was uncommon.

The incidence of NF hospitalizations rose by 2.7% per year (*P* = 0.0001), ranging from 5.7 to 7.7 hospitalizations per 100,000, with an overall rise of 35% between 2001 versus 2010. The overall incidence of NF was 6.9 hospitalizations per 100,000 person-years and rose with age, increasing from 0.75 to 13 hospitalizations per 100,000 person-years for age groups 15–19 years and those ≥ 65 years, respectively (*P* < 0.0001). Males had higher incidence of NF hospitalizations than females (6.7 versus 5.6 hospitalizations per 100,000 population-years, resp. (*P* < 0.0001)). The incidence of NF hospitalizations varied significantly across race/ethnicity groups, being lowest among whites (5.8 hospitalizations per 100,000 person-years), and was 6.9 and 8.9 hospitalizations per 100,000 person-years among Hispanics and blacks, respectively (*P* < 0.0001 for each versus whites and between themselves).

Other (non-NF) sites of infection were reported in 4116 (33.8%) NF hospitalizations. The most common reported sites included the intra-abdominal (10.9%), urinary (10.5%), and respiratory (6.3%). The key changes of epidemiological, demographic, clinical, resource utilization, and outcome features among NF hospitalizations between 2001-2002 and 2009-2010 are outlined in Table 2. At the end of the last decade, NF hospitalizations had more often one or more chronic comorbidities. The rate of diabetes among NF hospitalizations increased from 46% to 50% (*P* = 0.0027) and reported obesity rose more than 3-fold during study period. There was no significant change in reported HIV infection or drug or ethanol abuse (data not shown). On the other hand, NF hospitalizations included less commonly those aged 65 years or older. Lack of health insurance tended to increase from 17% to 19.2% between 2001-2002 and 2009-2010 (*P* = 0.0551).

One or more organ failures were reported in 40% of NF hospitalizations, involving most commonly the respiratory (21.3%), renal (20.8), and cardiovascular (16.1%) systems. The rates of all examined organ failures increased substantially over the past decade. The rate of NF hospitalizations with 1 or more failing organs nearly doubled from 28.5% to 51.7% between 2001-2002 and 2009-2010, respectively (*P* < 0.0001), while the rates of those with ≥3 organ failures rose more than 3-fold (*P* < 0.0001). One or more organ failures occurred more commonly among NF hospitalizations with chronic comorbidities than those without (41.8% versus 35.5%, resp.; *P* < 0.0001). An amputation was required in 472 NF hospitalizations (3.9%). The rate of amputation rose nearly 2-fold between 2001-2002 and 2009-2010, increasing from 2.9% to 5.1%, respectively (*P* = 0.0002).

ICU admission was required in 50.3% of NF hospitalizations. The rate of ICU admission increased between 2001-2002 and 2009-2010 from 43.6% to 55.5%, respectively (*P* < 0.0001). The use of invasive mechanical ventilation, central venous catheterization, and hemodialysis among NF hospitalizations increased overall and rose substantially among those with specific organ failures (with the exception of new hemodialysis for the latter) between 2001-2002 and 2009-2010: (a) mechanical ventilation: 57.9% versus 65.1% (*P* = 0.0334) among those with respiratory failure; (b) central venous catheterization: 31.3% versus 57% (*P* < 0.0001) among those with cardiovascular failure; (c) new hemodialysis: 6.1% versus 5.5% (*P* = 0.8589) among those with acute renal failure. Hyperbaric oxygen therapy was used in 300 NF hospitalizations (2.5%), with use decreasing between 2001-2002 and 2009-2010 from 3.0% to 1.3%, respectively (*P* < 0.0001). Hospital length of stay decreased by 0.9%/year during study period (*P* = 0.0063). However, the median, inflation-adjusted hospital charges increased annually by 3.8% (*P* = 0.0002), an increase of 37% between 2001-2002 and 2009-2010 (*P* < 0.0001).

Hospital mortality did not change significantly over study period (*P* = 0.5922), while mortality rate rose by 21% between 2001-2002 and 2009-2010 (*P* = 0.0428). The disposition destination of NF hospitalizations changed substantially between 2001-2002 and 2009-2010, with routine home discharge decreasing by nearly 30% (*P* < 0.0001), while discharge to long-term care facilities rose nearly 2.5-fold (*P* < 0.0001).

The predictors of hospital mortality among NF hospitalizations on multivariate logistic regression analyses are detailed in Table 3. Older age, lack of health insurance, Medicare and Medicaid insurance, presence of selected chronic comorbidities, management at a teaching hospital, presence of most types of organ failure, increasing number of failing organs, and need for mechanical ventilation or hemodialysis were associated with increased risk of death. The following covariates were associated with reduced risk of hospital mortality: male gender, obesity, tobacco use, diabetes, use of central venous catheterization, and use of hyperbaric oxygen. Patients' race/ethnicity, level of poverty and median income at the zip code of residence, and abuse of alcohol or drugs were not significantly associated with increased risk of death, once controlled for remaining covariates.

## 4. Discussion

We found that the incidence of NF hospitalizations rose substantially over the past decade when examined in a large population-based cohort. Chronic comorbidity was reported in most hospitalizations and there was increased severity of illness over time. NF hospitalizations in our cohort required increasingly admission to an ICU and had increasing requirement of life-support interventions. NF required prolonged hospitalization with rising hospital charges. Hospital mortality was relatively low in the present cohort and did not change over time. However, hospital survivors likely sustained increasing residual morbidity, with only about 1 in 4 having a routine home discharge at the end of the last decade.

Although several recent population-based studies were reported on necrotizing soft tissue infections [13, 14], the present study is, to our knowledge, the first population-level examination of contemporary trends of NF epidemiology in the United States (US). Our findings of rising NF incidence are higher than prior reports. A commonly cited multistate incidence estimate of NF in the US is 4 per 100,000, based on a report by Simonsen et al. [15], using administrative data from 1997 to 2002, with similar case definition. Markedly lower incidence of NF was found by Mulla et al. in another population study, using similar approach, with NF reported in 1.3 per 100,000 hospital discharges in Florida in 2001 [6]. However, the investigators focused only on NF as primary diagnosis. In a recent report from New Zealand by Das and colleagues, the investigators found rising incidence of NF from 0.18 to 1.69 per 100,000 from 1990 to 2006 [16]. The sources of the significant difference between our findings and those by Das et al. are not readily apparent, but they may be related in part to different population mix and a small sample size in the latter study. In addition, marked regional variations, as high as 10-fold, in the incidence of NF were noted by other investigators [3], though the sources remain unclear. Further studies are needed to corroborate our findings.

Several possible explanations may be considered for the apparent rise of incidence of NF in our cohort. Our findings may reflect increasing diagnosis of less severe skin and soft tissue infections as NF over time. However, this explanation is not supported by the evidence of rising severity of illness, matched by trends in use of life-support interventions, increased rates of ICU admission, rising hospital charges, and lack of decrease in case fatality.

Increased clinician awareness of a specific clinical condition should be considered as an alternative source of an apparent rise in its incidence. However, this explanation is less likely in the case of NF, as it remains a very rare diagnosis, as evidenced in the current study, with NF reported in about 0.05% of hospitalizations, with most clinicians in the state rarely encountering a NF patient. Our findings of rising NF incidence are in line with those by Das et al. [16] and Kaul et al. [17], which were based on direct record reviews. It may thus be hypothesized that the present findings reflect actual rise in the incidence of NF in the state. There are several possible explanations for rising incidence of NF in our cohort. Chronic comorbidities, and especially diabetes, well known to increase risk of NF, were increasingly present at the end of study period. In addition, obesity, a well-known risk factor for NF [1], was increasingly noted. It is likely that the rate of obesity, while rising in the general population [18], was underreported in our cohort, as can be the case in administrative data sets [19]. In addition, nearly 1 in 5 NF hospitalizations in the present cohort lacked health insurance, with increasing trend over the past decade, being more than 2-fold higher than the average among hospitalizations in the state [20] and comparable to a recent report by Holena et al. [7]. It is plausible that lack of regular and timely access to health care at the early phases of infection may have contributed to the observed trends in our study, though this would not explain similar trends noted by Das et al. [16] and Kaul et al. [17]. Contributing factors proposed by other investigators included changes in virulence and infectivity of infecting microorganisms [16], though the latter cannot be inferred through interrogation of administrative data sets. Additional studies in other states and nationally are warranted to further elucidate the evolving epidemiology of NF.

We found higher incidence of NF hospitalizations among the non-white population, similar to those reported by Das et al. [16]. However, the latter examined mostly Pacific Islander population. Nevertheless, the increased incidence of NF hospitalizations over the past decade in our study was predominantly driven by NF events among whites, rising by nearly 50%. The sources of differential incidence of NF and its trends across race/ethnicity groups require further investigation. Our findings of higher incidence of NF hospitalizations among males are in line with reports from New Zealand [16] and Canada [17] and may reflect in part higher risk of sepsis among males [21]. The majority of NF hospitalizations had one or more chronic comorbidities, with predominance of diabetes. These findings are similar to prior reports [2, 7] and may explain in part the relatively young age of the majority of affected patients.

An additional site of infection was common among NF hospitalizations, reported in about 1 in 3, mostly involving intra-abdominal sites and the urinary tract. It is unclear whether the reported infections preceded, followed, or occurred synchronously with NF. Infections of other sites complicating the course of NF were reported in 76% of an Australian cohort reported by Widjaja and colleagues [4], with urinary tract infections being the most common. The limited data on non-NF infections preclude assessment of their impact on the outcomes of NF patients.

Necrotizing fasciitis is commonly considered to be a critical illness, with reports often focused on patients managed in the ICU [4, 22]. We found that half of NF hospitalizations required ICU care, rising over the past decade. The pattern of ICU utilization among NF patients at the population level in the US has not been previously described. However, our findings are similar to those reported by Tillou and colleagues in the US, describing ICU admission in 61% of their patients with necrotizing soft tissue infections [23]. The latter results are comparable to reports on NF in Australia [4] and New Zealand [24], showing need for ICU care in 63% and 56% of their patients, respectively. Nevertheless, critical care utilization patterns can vary across countries [25] and regionally [26], limiting a direct comparison. Importantly, focus only on ICU-managed NF can underestimate the burden of NF in the population.

The respiratory, renal, and cardiovascular systems were the most commonly involved with OF in the present study. Das and colleagues reported acute renal failure, shock, and “coagulation abnormality” of 42%, 43%, and 22%, respectively, among their NF patients, with multiorgan failure in 18% [24]. However, the investigators did not define specific organ failures or multiorgan failure and did not describe trends of occurrence of failing organs over study period. We found organ failures more commonly among NF hospitalizations with chronic comorbidities, likely reflecting increased susceptibility of chronically ill patients to deterioration with NF. Our findings of rising rates of reported individual organ failures likely reflect actual increase in severity of illness, rather than upcoding by clinicians. This hypothesis is supported by increasing or unchanged rates of use of related life-support interventions, rising rates of admission to the ICU, and marked decrease in rates of routine home discharge, with more than doubling of rates of discharge to long-term care facilities. It is likely that increasing occurrence (and, possibly, severity) of chronic comorbidities over time in our cohort has contributed to higher severity of illness, as defined by increasingly reported individual and increased number of failing organs. However, as noted earlier, we cannot exclude the role of lack of health insurance in many NF hospitalizations, as well as possible increased virulence of infecting pathogens. Further studies are required to examine the sources of rising rates of organ failure among NF patients and to corroborate our findings.

Amputation was required in 3.9% of NF hospitalizations, comparable to 5.4% in a recent population-based study by Holena and colleagues [7]. Reported rates of amputation ranged from none [27] to 25.7% [28] in small cohorts. Amputation rates rose substantially over the last decade, possibly reflecting the increased severity of illness among NF hospitalizations. Nevertheless, most contemporary NF patients can be managed without need for amputation.

Hyperbaric oxygen therapy (HBO) was rarely used in the present cohort. Our findings are lower than those reported by Mulla and colleagues [6], who found use of HBO in 8.8%. However, the investigators examined a markedly smaller NF cohort (*n* = 216). Our study is the first, to our knowledge, to report on population-level trends of use of HBO in NF. We found that while the severity of illness rose over the past decade, use of HBO declined among NF hospitalizations. However, the sources of this trend are unclear and further studies are required to corroborate our findings.

More than 1 in 4 NF hospitalizations received one or more of the examined life-support interventions, with increased utilization rates at the end of the last decade, congruent with rising occurrence of organ failure. Use of life-support interventions was generally not reported in population-based studies of NF. Das and colleagues reported use of dialysis in 8.5% of their patients in New Zealand [24]. Bucca et al. described use of mechanical ventilation and continuous renal replacement therapy in 71% and 29%, respectively, among 24 NF patients [22]. However, the investigators restricted their report to ICU patients, and additional study is warranted on the use of life-support interventions in other NF populations.

Hospital length of stay among NF hospitalization was nearly 3.5-fold longer than the average in the state [20], reflecting patients' severity of illness. Our findings are comparable to prior population-based reports on NF in the US [6] but are markedly shorter than mean length of stay in Canada (33.3 days) [3] and Australia (36 days) [4], likely reflecting in part variation of practice patterns. The fiscal burden of NF in the present cohort in 2001 was somewhat lower than that reported by Mulla and colleagues (median hospital charges $59,399 versus $69,146, resp. (2010 dollars)) [6]. The source of the difference may reflect different patient and payor mix, care processes, and smaller sample size in the latter study. However, the average hospital charges for NF hospitalizations in our cohort in 2009 ($145,165) exceeded the most expensive hospitalization condition in the state (respiratory failure ($103,152); all in 2010 dollars) [20]. Widjaja and colleagues reported the average costs of managing NF in Australia ($55,867 in 2010 dollars) [4]. However, the latter findings cannot be readily compared to those in the US. We found marked increase in hospital charges over the past decade, likely related to increased severity of illness. However, there was a small but statistically significant decrease in hospital length of stay, possibly reflecting improved care efficiencies and increased discharge to long-term care facilities, which more than doubled during study period. Thus, NF is associated with ongoing, high hospital resource utilization burden.

Hospital mortality was relatively low among NF hospitalizations in the state, similar to that reported in the population-based studies, ranging from 9.3% [7] to 11.1% [6], suggesting decline of hospital mortality among contemporary NF hospitalizations in the US. Hospital mortality tended to be as high as 33% in local studies in the US [29] and ranged from 11.1% [4] to 23.5% [24] in Australia and New Zealand, respectively. There have been, to our knowledge, no recent reports on the population-level trends of case fatality of NF in the US. The rising mortality rate in our cohort most likely reflects the rising incidence of NF.

The unchanged, rather than rising, hospital mortality may represent improved outcome, considering the rising severity of illness, as judged by increased occurrence and number of organ failures. However, for proper context, hospital mortality has been decreasing among patients with severe sepsis over the same period, despite similarly rising development of organ failure [11], possibly in part due to increased clinician awareness and overall improved care. On the other hand, NF remains rare, with most physicians not seeing even a single patient in a given year or longer, affecting early recognition and timely care. In addition, it is conceivable that the lack of change in hospital mortality in the present cohort may rather underestimate trends of short-term mortality due to change in location of patients' death. This hypothesis is supported by more than double rate of discharges to long-term care facilities among hospital survivors, supporting, as noted earlier, higher residual morbidity. Indeed, a recent report by Reineck and colleagues documented that a marked variability in adjusted hospital mortality is a function of a “discharge bias” related to varying discharge practices [30], corroborating an earlier study by Hall et al. who noted that transfers to long-term acute care facilities can explain a significant proportion of the variation of reported hospital mortality [31].

Rising age and presence of selected comorbid conditions were associated with increased risk of hospital mortality, similar to reports by other investigators [2, 3, 7, 17, 28]. Our findings of reduced risk of death among males with NF contrast the lack of gender-related prognostic impact reported by others [6, 7, 28]. The sources of the observed difference in our cohort cannot be inferred due the observational, retrospective design of our study and inability to exclude residual confounding. However, unlike previous studies, we have controlled our prognostic models for the type and number of organ failures and life-support interventions, which may have affected our findings. Given the conflicting data on the role of gender on patient outcomes in severe infections, further studies are warranted on its role in NF. Patients' race did not affect mortality risk, once controlled for other confounders, similar to the report by Holena and colleagues [7], though contrasting the adverse impact of race in severe sepsis [32]. However, the mechanism underlying racial differences of infection-related epidemiology observed by others remains elusive [32]. Lack of health insurance was independently associated with higher risk of death, in line with other reports [7], likely reflecting in part barriers for regular and timely access to health care. The adverse prognostic association of management in a teaching hospital, noted by others [7], is likely the function of the higher-risk population commonly managed in teaching facilities.

The prognostic role of failing organs or need for life-support interventions in NF has not been previously examined, to our knowledge. We found that the development of most types of organ failure, rising of their number, and need for invasive mechanical ventilation and hemodialysis were all associated, as expected, with increased risk of death. The adverse impact was especially high with respiratory and cardiovascular organ failures. Our finding of a “protective” effect of central venous catheterization may reflect a subset of NF hospitalizations with more effective circulatory support or overall care. However, our study design precludes direct inference about the source of this finding.

The prognostic effect of HBO in NF remains unsettled in the absence of controlled trials and with mixed results of small observational reports [1]. Use of HBO was associated with the highest reduction in mortality risk in our cohort. In the other population-based studies of NF, the prognostic impact of HBO either was not examined [7] or was not significant [6]. However, as noted earlier, HBO was used only in 19 NF hospitalizations in the latter study [6], precluding meaningful analysis. Examination of the impact of HBO on NF outcomes awaits a controlled trial and, given the associated challenges, warrants further study in other populations.

Obesity was associated with reduced odds of death among NF hospitalizations, though its prognostic role was not previously examined in this population. Recent data have suggested a protective role of obesity among critically ill patients [33] in general and among those with severe sepsis [34]. However, a recent study by Robinson and colleagues demonstrated lack of independent prognostic impact of obesity, once adjusted for patients' nutritional state [35], which cannot be examined in administrative data sets.

The sources of the observed “protective” association between diabetes and patients' mortality in the present cohort remain uncertain. Although diabetes can increase the risk of infection and is a well-known risk factor for NF, findings on its prognostic impact have been inconsistent. Several studies found increased risk of infection-associated mortality among diabetic patients [36, 37]. However, more recent reports described lower case fatality [38] and lower adjusted odds of death [39] in septic patients with diabetes. It has been suggested that the differential effect of diabetes on development of sepsis-related organ failure, including lower risk of respiratory failure, as compared with nondiabetic patients [38], may affect mortality risk of the former. However, the underlying mechanisms of the complex interplay between the chronic effects of diabetes and its state of control and the acute response of diabetic patients to an infectious insult remain to be elucidated.

Smoking history had an unexpectedly “protective” effect on the risk of hospital death. While being clearly associated with increased risk of skin and soft tissue infections [40], there are no specific data on the morality impact of smoking in these infections [40]. Of note, although smoking is considered a risk factor for pneumonia, it was not associated with poor outcomes in a multinational study of bacteremic pneumococcal disease [41]. The prognostic role of smoking in NF has not been previously examined, and we cannot exclude residual confounding as source of our findings.

Despite the use of a large, longitudinal, and well-characterized database, our findings should be considered in the context of several limitations. First, retrospective design and use of an administrative data set with their attendant limitations affect the interpretation of our results. However, the rarity of NF limits population-level approaches to study this condition. In addition, the deidentified data do not allow accounting for multiple hospitalizations by the same patient during specific period. However, similar approach with the aforementioned limitations was used by other investigators of NF [6, 7].

The case definition of NF in the present study has been based on ICD-9-CM coding at reporting hospitals. Administrative data sets do not provide information on pathological confirmation of NF diagnosis, raising a potential for misclassification. Nevertheless, NF diagnoses were reported very sparingly (0.05%) in our cohort and it is unlikely that miscoding occurred systematically or incrementally over time and thus misclassification is unlikely to explain the rise in NF incidence. In addition, the morbidity burden of NF in the present study, as judged by rate of ICU admission and hospital length of stay, is comparable to other reports on NF [4, 6, 24], including those based on review of medical records. On the other hand, we cannot exclude underestimation of NF in our cohort. Finally, our case identification approach is similar to prior population-level reports of NF [6].

The administrative data set used in the present study did not provide specific reports on the anatomical sites affected by NF. Thus, the prognostic impact of site-specific NF could not be explored. Similar constraints have affected prior population-based studies [6, 7].

The use of administrative data in our study precluded access to information on the timeliness of diagnosis of NF and to details, time course, and appropriateness of antimicrobial therapy, direct source control, and resuscitative interventions, all of which may vary across institutions and individual clinicians and likely have affected the observed resource utilization and outcomes. However, as noted earlier, similar constraints affect interpretation of prior population-level studies of NF [6, 7]. Because the state of Texas does not provide tools to convert hospital charges to costs, we reported hospital charges rather than costs of care, limiting comparisons with other cost data. However, the available charge data allowed comparisons within state population.

Gender and zip code masking among hospitalizations with diagnoses of HIV infection and drug or ethanol abuse restricted the assessment of the prognostic role of gender and local economic state in this subgroup. However, the aforementioned diagnoses accounted for less than 10% of our cohort. Finally, although we have performed extensive adjustment for confounders in our predictive models, we cannot exclude residual, unaccounted confounding that may have affected our results.

## 5. Conclusions

We report the largest population-level study to date of NF, describing progressive rise in its incidence and severity over the past decade. The majority of NF hospitalizations required care in an ICU by the end of the last decade, with common use of life-support interventions. NF patients required prolonged hospitalization with high hospital charges, making NF possibly the costliest hospital diagnosis in the state. Hospital mortality remained relatively low but may have underestimated short-term mortality, while NF was likely associated with substantial residual morbidity among hospital survivors. Further studies of NF are needed in other populations to corroborate our findings.

## Supplementary Material

The following tabular data provide a detailed description of the International Classification of Diseases, Ninth Edition, Clinical Modification codes utilized to derive data on sites of infection, failing organ systems, and selected life-support procedures.

## Figures and Tables

**Table 1:** Characteristics of NF hospitalizations.

Group	*n* = 12,172
Age [years, *n* (%)]	
15–64	9,226 (75.8)
65–74	1,668 (13.7)
≥75	1,278 (10.5)
Gender [*n* (%)]^a^	
Female	5,036 (46)
Male	5,922 (54)
Race/ethnicity [*n* (%)]	
Hispanic	4,138 (34)
Black	1,826 (15)
White	5,356 (44)
Other	852 (7)
Health insurance [*n* (%)]	
Private	3,980 (33)
Medicare	4,008 (33)
Medicaid	1,408 (12)
Uninsured	2,354 (19)
Other	417 (4)
Missing	5 (<1)
Chronic comorbidities [*n* (%)]^b^	
Any	8,793 (72.2)
Congestive heart failure	1,557 (12.8)
Peripheral vascular disease	1,239 (10.2)
Chronic pulmonary disease	1,368 (11.2)
Diabetes mellitus	5,794 (47.6)
Dementia	90 (0.7)
Myocardial infarction	439 (3.6)
Connective tissue disease	287 (2.4)
Cerebrovascular disease	474 (3.9)
Chronic renal failure	1,882 (15.5)
Malignancy	578 (4.7)
Chronic liver disease	786 (6.5)
HIV^c^ infection	74 (0.6)
Deyo-Charlson score	
mean (SD)	1.8 (1.9)
Obesity [*n* (%)]	1,855 (15.2)
Drug abuse [*n* (%)]	586 (4.8)
Alcohol abuse [*n* (%)]	628 (5.2)

^a^Gender was masked in 1214 hospitalizations. The denominator used to derive female/male percentage for the cohort was based on hospitalizations with available gender designation (*n* = 10,958); ^b^based on the Deyo-Charlson comorbidity index; ^c^human immunodeficiency virus.

**Table 2:** Changes in the epidemiology, patient characteristics, resource utilization, and outcomes of NF hospitalizations.

Variable	2001-2002 (*n* = 1,976)	2009-2010 (*n* = 2,926)	*P *
Age-adjusted incidence (per 10^5^ population/yrs)			
All	5.9	7.6	<0.0001
Hispanic	7.1	7.1	0.9203
Black	8.3	8.9	0.4140
White	4.7	7.0	<0.0001
Other	9.8	14.3	0.0008
Age ≥65 years (*n*, %)	540 (27.3)	694 (23.7)	0.0048
Any chronic comorbidity (*n* [%])^a^	1,371 (69.4)	2,244 (76.7)	<0.0001
Deyo-Charlson score [mean (SD)]	1.6 (1.7)	2.1 (2)	<0.0001
Obesity (*n*, %)	139 (7)	677 (23.1)	<0.0001
Number of failing organs			
Median (IQR)	0 (0-1)	1 (0–2)	<0.0001
≥3 Organ failures (%)	117 (5.9)	534 (18.3)	<0.0001
Organ failures (*n* [%])			
Respiratory	299 (15.1)	776 (26.5)	<0.0001
Cardiovascular	192 (9.7)	686 (23.4)	<0.0001
Renal	214 (10.8)	949 (32.4)	<0.0001
Hepatic	14 (0.7)	85 (2.9)	<0.0001
Hematological	128 (6.5)	338 (11.6)	<0.0001
Metabolic	96 (4.9)	340 (11.6)	<0.0001
Neurological	24 (1.2)	197 (6.7)	<0.0001
Life-support interventions (*n* [%])			
Mechanical ventilation	173 (8.8)	505 (17.3)	<0.0001
CVC^b^	362 (18.3)	1088 (37.2)	<0.0001
Hemodialysis	45 (2.3)	136 (4.6)	<0.0001
New hemodialysis	13 (0.7)	52 (1.8)	0.0012
Hospital length of stay, days			
Median (IQR)	14 (7–25)	13 (7–25)	0.2911
Mean (SD)	19.3 (18.6)	19.1 (19.7)	
Total hospital charges, dollars^c^			
Median (IQR)	65,808 [34,036–135,468]	90,460 [46,278–167,262]	<0.0001
Mean (SD)	115,635 (146,549)	142,683 (179,948)	
Mortality rate (per 10^5^ population)	0.61	0.74	0.0428
Patient disposition (*n* [%])			
Hospital mortality	203 (10.3)	287 (9.8)	0.6287
Routine discharge	791 (40)	822 (28.1)	<0.0001
Home health care	313 (15.8)	668 (22.8)	<0.0001
Short-term facility	323 (16.3)	200 (6.8)	<0.0001
Long-term facility	242 (12.2)	879 (30)	<0.0001
Other^d^	31 (1.6)	55 (1.9)	0.4825
Missing	73 (3.7)	15 (0.5)	<0.0001

^a^Based on the Deyo-Charlson comorbidity index; ^b^central venous catheterization;^ c^hospital charges are adjusted for inflation to 2010 dollars; ^d^left against medical advice, hospice.

**Table 3:** Predictors of hospital mortality among hospitalizations with necrotizing fasciitis.

Covariate	Odds ratio	95% confidence interval	*P*
Age (years)^a^			
65–74	1.491	1.128–1.972	0.0050
≥74	2.256	1.763–2.888	<0.0001
Male^b^	0.802	0.681–0.945	0.0083
Health insurance^c^			
No health insurance	1.527	1.176–1.983	0.0015
Medicaid	1.302	0.993–1.707	0.7613
Medicare	1.666	1.324–2.097	0.0259
Smoking	0.603	0.439–0.827	0.0017
Alcohol abuse	1.165	0.873–1.556	0.2993
Obesity	0.582	0.444–0.762	<0.0001
Chronic comorbidity			
Myocardial infarction	1.414	1.009–1.981	0.0441
Congestive heart failure	1.695	1.387–2.071	<0.0001
Dementia	1.485	0.725–3.041	0.2798
Diabetes	0.750	0.623–0.903	0.0023
Chronic renal disease	1.738	1.424–2.121	<0.0001
Chronic liver disease	3.204	2.336–4.394	<0.0001
Malignancy	2.577	1.934–3.434	<0.0001
Cerebrovascular disease	1.128	0.776–1.639	0.5288
HIV infection^d^	1.524	0.721–3.225	0.2700
Teaching hospital	1.208	1.014–1.438	0.0344
Type of organ failure			
Respiratory	3.392	2.679–4.294	<0.0001
Type of organ failure			
Cardiovascular	2.820	2.336–3.406	<0.0001
Renal	1.308	1.088–1.573	0.0042
Hepatic	0.797	0.492–1.290	0.3561
Hematological	1.597	1.288–1.980	<0.0001
Metabolic	1.553	1.250–1.930	<0.0001
Neurological	1.661	0.800–3.451	0.1735
Number of organ failures^e^			
1 organ failure	3.148	2.475–4.005	0.0001
2 organ failures	6.650	5.163–8.565	<0.0001
≥3 organ failures	13.500	10.471–17.406	<0.0001
Life-support interventions			
Mechanical ventilation	1.851	1.467–2.335	<0.0001
Central venous catheterization	0.742	0.620–0.888	0.0011
Hemodialysis	1.337	1.020–1.754	0.0354
Hyperbaric oxygen	0.114	0.026–0.495	0.0037

^a^Age < 65 years was used as referent; ^b^female gender was used as referent; gender was masked in 1214 hospitalizations. The model to examine the role of gender was restricted to hospitalizations with available gender designation (*n* = 10,958); ^c^private insurance was used as referent; ^d^human immunodeficiency virus/acquired immunodeficiency syndrome; ^e^hospitalizations with zero organ failures were used as referent.
