# Supplementary material for: Contemporary Trends of the Epidemiology, Clinical Characteristics, and Resource Utilization of Necrotizing Fasciitis in Texas: A Population-Based Cohort Study
Source: Crit Care Res Pract. 2015 Mar 29;2015:618067. doi: 10.1155/2015/618067 (PMC4393891; doi:10.1155/2015/618067)
Supplement: Supplementary file 1 — The following tabular data provide a detailed description of the International Classification of Diseases, Ninth Edition, Clinical Modification codes utilized to derive data on sites of infection, failing organ systems, and selected life-support procedures. [file 618067.f1.docx]

**Supplementary Table 1.** International Classification of Diseases, Ninth Edition, Clinical Modification (ICD 9 -CM) codes for sites of infection (Where only 3 or 4-digit codes are listed, all associated subcodes are included.)

Category ICD-9-CM codes

Respiratory 481-486, 510, 513

Blood 790.7, 572.1, 673.3

Endocarditis 112.81, 421

Central nervous system 320, 322, 324, 325

Gastrointestinal/abdominal 003, 008, 540-542, 530.4, 530.86, 562.01, 562.03, 562.11, 562.13, 566, 567, 569.5, 569.83, 572.0, 575.0, 531.1, 531.2, 531.5, 531.6 532.1, 532.2, 532.5, 532.6, 533.1, 533.2, 533.5, 533.6, 534.1, 534.2, 534.5, 534.6

Urinary 590, 599.0

Genital 615, 634.0, 635.0, 636.0, 637.0, 638.0, 639.0, 646.6 658.4

Skin and soft tissue 675.1, 680, 682, 686, 998.5

Bone and joint 711.0, 730

Device-related 996.6

**Supplementary Table 2.** International Classification of Diseases, Ninth Edition, Clinical Modification codes for classification of organ failure (Where only 3 or 4-digit codes are listed, all associated subcodes are included.)

Category ICD-9-CM codes

Respiratory

Acute respiratory failure 518.81

Other pulmonary insufficiency, not

elsewhere specified (includes

acute respiratory distress, acute

respiratory insufficiency, acute

respiratory distress syndrome) 518.82

Pulmonary insufficiency following trauma,

or surgery 518.5

Acute and chronic respiratory failure 518.84

Respiratory abnormalities, not otherwise

specified 786.09

Respiratory arrest 799.1

Invasive mechanical ventilation 96.7-96.72

Cardiovascular

Hypotension, not otherwise specified 458.8, 458.9

Shock, not otherwise specified 785.50

Cardiogenic shock 785.51

Other shock without mention of trauma 785.59

Renal

Acute renal failure 584

Hepatic

Acute necrosis of liver 570

Hepatic encephalopathy 572.2

Hepatic infarction 573.4

Hematologic

Defibrination syndrome 286.6

Acquired coagulation factor deficiency 286.7

Coagulopathy (Other unspecified

coagulation defects) 286.9

Thrombocytopenia (secondary or

unspecified) 287.4, 287.5

Metabolic

Acidosis (metabolic or lactic) 276.2

Neurologic

Acute and subacute delirium 293.0, 293.1

Anoxic brain damage 348.1

Encephalopathy, not elsewhere classified 348.3

Coma 780.01

Other alteration of consciousness 780.09

**Supplementary Table 3.** International Classification of Diseases, Ninth Edition, Clinical Modification (ICD-9-CM) codes for selected life-support procedures

Category ICD-9-CM codes

Mechanical ventilation 96.70-96.72

Central venous catheterization 38.96, 38.97

Hemodialysis 38.95
